# Medicinal plants used to control internal and external parasites in goats

**DOI:** 10.4102/ojvr.v83i1.1016

**Published:** 2016-04-29

**Authors:** Marcia Sanhokwe, Johnfisher Mupangwa, Patrick J. Masika, Viola Maphosa, Voster Muchenje

**Affiliations:** 1 Department of Livestock and Pasture Science, University of Fort Hare Alice, South Africa; 2 Fort Cox College of Agriculture and Forestry, Middledrift, South Africa

## Abstract

The use of medicinal plants plays a major role in the primary health care of animals in South Africa. A survey was conducted to document medicinal plants used to control parasites in goats in Kwezi and Ntambethemba villages in the Eastern Cape Province, South Africa. Information from 50 farmers and 3 herbalists was obtained through the use of a structured questionnaire, and a snowball sampling technique was used to identify key informants. The obtained data were analysed using PROC FREQ of SAS (2003), and fidelity level values were determined to estimate the healing potential of the mentioned plants. The survey revealed nine plant species belonging to eight families that were used to control parasites in goats. Asphodelaceae (22.22%) was the most frequently used plant family. Leaves were the most used plant parts, constituting 60.38%. They were prepared either as infusions or decoctions of single plants or in mixtures. *Aloe ferox, Acokanthera oppositifolia* and *Elephantorrhiza elephantina* were the plants having the highest fidelity level for their use to control parasites, each scoring 100%, followed by *Albuca setosa* (83.33%). The study revealed low knowledge about ethno-veterinary medicine in the study area. It also revealed that information on ethno-veterinary medicine in this area is mostly confined to older people and there is danger that this knowledge can be lost before being passed on to other generations. Therefore, there is an urgent need to document information on these plant species so that the future generation can benefit. Further investigation should be carried out to validate the efficacy and safety of the above-mentioned plants so as to provide cheap alternative ways of controlling parasites.

## Introduction

Goats play an important role in the socio-economic activities of people, especially in developing countries, by providing food and income (Peacock 2005). However, parasites limit goat productivity as they reduce fertility, cause skin irritation and suck blood, ultimately leading to death (Molefe *et al*. 2012). Gastrointestinal parasites such as *Haemonchus contortus* and *Fasciola hepatica* are a major health problem in small ruminants (Vatta & Lindberg 2006). External parasites such as ticks, lice and mites have also been reported in goats. Most of these parasites are more prevalent in developing countries because of inappropriate housing and lack of adequate veterinary services (Mungube *et al*. 2006). Commercial drugs are mostly used to control parasites; however, these are expensive and are out of reach for many resource-poor farmers. Some of the parasites have also developed resistance against these drugs (Clark, Stephen & Cawley 1996), and the drugs can pollute the environment (Wall 2007). This has led farmers to resort to alternative measures that include the use of medicinal plants to treat and control livestock parasites. There is also a belief that natural products are safe to use and harmonious with the biological system (Erasto 2003). Knowledge on the use of ethno-veterinary medicine is passed on orally, and there is a danger that this information might disappear because of technical and socio-economic changes. Therefore, this study was conducted to document the medicinal plants used to control internal and external parasites in goats in the Chris Hani District Municipality, South Africa. This will help in the pharmacological study of these plants and in the development of therapeutic drugs that have fewer side effects than synthetic chemicals.

## Materials and method

### Study site

The survey was conducted in Chris Hani District Municipality in two local municipalities, namely Emalahleni and Tsolwana, where one village each was randomly selected, namely Kwezi and Ntambethemba, respectively. The area lies within latitude 31°70’63–32°31’34S and longitude 27°23’41–27°51’17E. It receives an average annual rainfall of 483 mm, with most rain occurring in summer. The study area has an average minimum and maximum temperature of 7 °C and 22 °C, respectively (Institute for Soil, Climate and Water 2008). The area is covered by Eastern Mixed Nama Karoo, Sub-arid Thorn Bushveld, South-Eastern Mountain Grassland and Moist Bushland veld types (Acocks 1975).

### Sampling procedure

Villages were randomly selected, and farmers who keep goats were identified using the snowball sampling procedure. This sampling technique, which involved approaching goat farmers and herbalists with more knowledge on the plants used in treating internal and external parasites, in turn directed us to other potential respondents (Patton 1990). Interviews were conducted amongst 50 farmers and 3 herbalists.

### Data collection

Structured questionnaires were used to collect data. The data collected included demographic information such as gender, age, source of information and employment status. Information gathered included local name of plant used, condition of plant used (dry or fresh), plant parts used, method of preparation, adverse effects, source of knowledge, parasites affecting livestock, other methods used to control parasites, dosage and their source of knowledge. This survey was carried out in accordance with the University of Fort Hare Research and Ethics Policy (Ethical certificate number MAP011SSAN01). Plants were collected with the help of herbalists and were authenticated by a botanist, Professor Grierson at the University of Fort.

### Data analysis

Descriptive statistics were obtained using PROC FREQ of SAS (2003). Fidelity level (FL) values were determined to report the most used plants in the different communities, as this can be an indication of their possible efficacy (Table 1). This was calculated for plant species that had been reported more than three times. FL is the percentage of respondents who use a certain plant for the same main function (Sofowara 1982) and was calculated as:

$$(N_{a}/N)\times 100$$ [Eqn 1]

where *N_a_* is the number of respondents who claim the use of a plant species to treat a particular ailment and *N* is the number of informants who use the plant as medicine for any ailment (Alexiades 1996).

**TABLE 1:** Fidelity level indices of plant species used to control parasites in the study area.

Species	Parasite controlled	*Na*	*N*	FL in % [(Na/N) × 100]
*Aloe ferox*	Helminths, ticks, mites	23	23	100
*Acokanthera oppositifolia*	Helminths, ticks	8	8	100
*Elephantorrhiza elephantina*	Helminths, mites, ticks	6	6	100
*Albuca setosa*	Helminths	5	6	83.33
*Gunnera perpensa*	Helminths	3	9	33.33
*Centella coriacea*	Helminths	1	4	25
*Cussonia spicata*	Helminths	1	6	16.67

*Na*, indicates number of respondents who claim a use of a plant species to treat a particular ailment; *N*, indicates the number of informants who use the plant as medicine for any ailment; FL, Fidelity level.

## Results

### Demographic information

The demographic data of respondents are shown in Table 2. The majority of the households were male headed (73.58%). The most dominant age group within these heads was above 51 years (84.91%), followed by those aged 31–50 years (13.21%). Most of the respondents (43.40%) never went to school. It was found that most of the respondents were unemployed (71.70%) and depended on government grants (39.62%) and selling livestock (35.85%) as their source of income.

**TABLE 2:** Demographic data on distribution of respondents.

Demographic data	Variables	Proportion	%	Frequency	%
Age	20–30	1	1.89	-	-
	31–50	7	13.21	-	-
	≥ 51	45	84.91	-	-
	**Total**	**53**	**100**	**-**	**-**
Level of education	Primary	19	35.85	-	-
	Secondary	9	16.98	-	-
	Tertiary	2	3.77	-	-
	Never went to school	23	43.40	-	-
	**Total**	**53**	**100**	**-**	**-**
Employment status	Employed	3	5.66	-	-
	Unemployed	38	71.70	-	-
	Self-employed	2	3.77	-	-
	Retired	10	18.87	-	-
	**Total**	**53**	**100**	**-**	**-**
Source of income	Salary	10	18.87	-	-
	Livestock	19	35.85	-	-
	Crop farming	3	5.66	-	-
	Grant	21	39.62	-	-
	**Total**	**53**	**100**	**-**	**-**
Gender	Male	-	-	39	73.58
	Female	-	-	14	26.42
	**Total**	**-**	**-**	**53**	**100**

### Livestock inventory

All respondents owned livestock, which included cattle (92.45%), sheep (71.70%), goats (100.00%) and chickens (75.47%). Farmers’ reasons for keeping goats were for multiple purposes such as meat, milk, income and for cultural or religious reasons. Most of the farmers kept goats for cultural or religious reasons (58.49%). Most of the farmers had more than 40 sheep, whilst cattle, goats and chickens were kept in smaller numbers ranging from 1 to 10.

### Prevalence of diseases and parasites

Gallsickness, heartwater, redwater, diarrhoea and bloating are some of the diseases that were reported to affect goats in the area. The most prevalent tick-borne diseases in the area were heartwater (10.44%), redwater (11.54%) and gallsickness (60.02%). These diseases were more prevalent in summer. Government veterinary officers helped the farmers to identify the diseases, and this helped in providing the right treatment. Most of the farmers (88.68%) treated their goats when suffering from these diseases.

**FIGURE 1:**
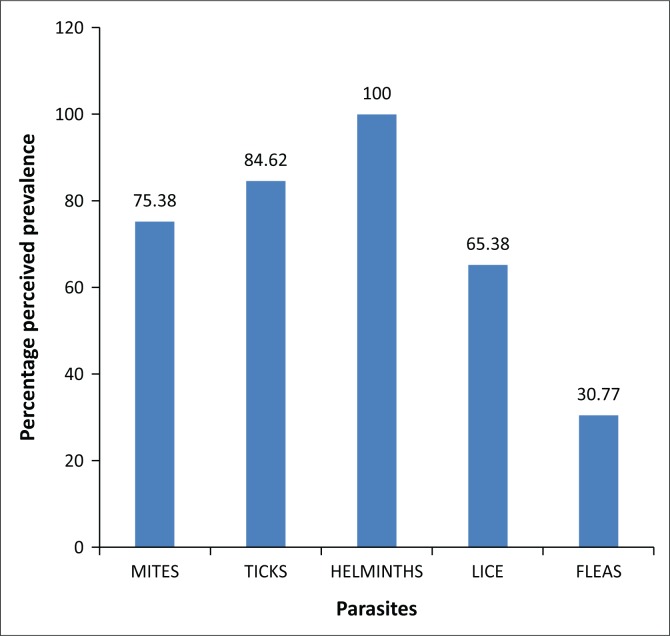
Perceived prevalence of parasites in Chris Hani District in Eastern Cape Province, South Africa.

All respondents acknowledged both internal and external parasites to cause huge problems. Diseases, parasites, stock theft and poor rangelands were reported as the main challenges faced by the farmers in livestock rearing. Parasites (47.17%) were reported as the most problematic challenge that the farmers are facing. Common parasites in the study area were fleas (30.77%), lice (65.38), mites (75.38%), ticks (84.62%) and helminths (100%), all of which were reported to be more prevalent in summer than any other season of the year. Figure 1 shows the prevalence of parasites in the study area. Farmers were able to tell that a goat had been infested with parasites through loss in body condition (32.08%), loss of appetite (33.96%) and rubbing against poles (33.96%).

### Parasite control

The study revealed that most of the farmers (96.23%) dipped their goats once a month. The government provided commercial drugs for use in a spray race or dip tanks. Dazzle dip (Diazinon 30%) was one of the commercial drugs that they used to control external parasites in the area. Dipping was carried out once a month. Those farmers who did not dip (3.77%) felt there was no need to do that as they believed that goats are resistant to parasites. The majority of the respondents (69.23%) used medicinal plants, a few used commercial drugs (11.54%), and a proportion (19.23%) used both medicinal plants and commercial drugs to control parasites in their herd. Farmers used medicinal plants for various reasons, that is, they are effective (69.81%), cheap (1.89%), easily accessible (13.21%) and easy to use (15.09%), whilst others (11.54%) indicated that they did not have knowledge about the plants.

Overall, nine plants belonging to eight families used to control parasites in goats were reported as shown in Table 3. Asphodelaceae was the most frequently mentioned plant family (22.22%). *Aloe ferox* was the most used plant in the area (43.40%). Different plant parts such as leaves, roots, tubers and bark were used in preparing the remedies. Most of the farmers used leaves (60.38%) in preparing the medicine. For their preparation method, (60.38%) respondents used decoctions and (39.62%) infusions. No side effects were reported by the respondents. Some of the respondents combined more than one plant in the preparation of medicines. Others also mixed plant extracts with nonplant materials such as Epsom salts, flour, butter, potassium permanganate, rock salt and oil cakes. FL values were determined so as to estimate the medicinal use values and the relative preference of species by the local communities in this study area. Accordingly, *A. ferox, E. elephantina* and *Acokanthera oppositifolia* were the plants having the highest FL values for their use to control parasites, each scoring 100.00%, followed by *A. setosa* (83.33%). Most of the respondents (75.47%) reported that they acquired their knowledge orally from their parents and from other farmers. About 88.68% of respondents did not put any conservation methods in place to prevent plants from becoming extinct. The reasons were that there is not enough land to cultivate the plants (50.94%), and 49.06% of the respondents believed the plants are abundant in the wild and there is no need to conserve them. Those who practised conservation cultivated the plants in their gardens and used plant parts such as leaves, which does not destroy the whole plant.

**TABLE 3:** Plants used to control parasites in goats.

Family	Scientific name	Local name	Voucher number	Plant part used	Preparation method	Dosage	Type of parasite controlled
Asphodelaceae	*Aloe ferox*	Ikhala elikhulu	MSAN01/2015	Leaves	Infusion	Leaves are crushed and the juice is applied to the skin or mixed with drinking water	Helminths, ticks, mites
Fabaceae	*Elephantorrhiza elephantina*	Intolwane	MSAN02/2015	Roots	Decoction	Grind the roots and boil in water for about 30 minutes until the water turns red. Dose 300 mL or spray the animals	Helminths, mites, ticks
Hyacinthaceae	*Albuca setosa*	Ingwebeba	MSAN03/2015	Tuber	Decoction	Crush the tuber, boil and dose with a 500-mL bottle	Helminths
Apocynaceae	*Acokanthera oppositifolia*	Intlungunyemba	MSAN04/2015	Leaves	Decoction	Grind leaves, boil, cool and drench the animals. Dose with 1-L bottle for adults and a 300-mL bottle for kids	Helminths, ticks
Apiaceae	*Centella coriacea*	Inyongwana	MSAN05/2015	Bark	Decoction	Chop the bark, make a decoction, sieve and dose approximately 500 mL	Helminths
Araliaceae	*Cussonia spicata*	Umsenge	MSAN06/2015	Bark	Infusion	Grind the bark, soak overnight and dose 300 mL	Helminths
Gunneraceae	*Gunnera perpensa*	Iphuzi	MSAN08/2015	Tuber	Decoction	Crush the tuber, boil and dose 300 mL	Helminths
Agapanthaceae	*Agapanthus praecox*	Umkondo	MSAN09/2015	Leaves	Infusion	Grind the leaves, soak in water overnight and dose 500 mL	Helminths
Asphodelaceae	*Bulbine latifolia*	Ingcelwana	MSAN07/2015	Leaves	Decoction	Grind leaves, boil and apply to skin or drench with 1 L	Ticks, helminths

## Discussion

In this study the demographic characteristics of the farmers were similar to those reported by Mwale and Masika (2009) in the Eastern Cape and Limpopo Provinces of South Africa (Luseba & Van der Merwe 2006). The majority of the households were male headed, whilst the most dominant age group consisted of older generations. Most of the respondents who were using medicinal plants and had essential knowledge were generally older than 51 years. The findings of this study agree with those of Wanzala *et al*. (2005), who mentioned that information on medicinal plants is mostly stored in the memory of a few older people entrusted with it within communities. Most of these older people are unemployed and rely on grants for their survival. This concurs with findings by Masika, Van Averbeke and Sonandi (2000) who reported that most of the farmers rely on grants. The reason could be that most of them are old and poor, so grants are their major source of income.

The reasons for keeping goats were similar to those cited by Bester, Ramsay and Scholtz (2009) and Masika and Mafu (2004). Most of the farmers were keeping goats for cultural reasons and for cash income. The use of goats for milk was unpopular as reported previously by Mahajana and Cronje, (2000) and Masika and Mafu (2004). Furthermore, most of the farmers mentioned parasites as the most problematic challenge that they were facing and this concurs with studies by Mwale and Masika (2009). Parasites might be a problem in this area because the animals are raised on poorly managed pastures where parasites are abundant. Infestation with internal and external parasites was reported to be more prevalent in the summer season, which could be because of poor management of pastures (Masika & Mafu 2004), inappropriate housing (Mungube *et al*. 2006) and climatic conditions in the tropics (Webb & Mamabolo 2004; Phiri *et al*. 2007), which are favourable for parasite development.

Findings from this study revealed that farmers usually dipped their goats to control parasites. However, this contradicts the findings by Kunene and Fossey (2010), who observed that most goats are hardly dipped in rural communities. The farmers questioned in this study are not provided with commercial drugs to control internal parasites. The majority of the respondents (69.23%) used medicinal plants, whilst others bought their own commercial drugs (11.54%) and a proportion (19.23%) used both medicinal plants and commercial drugs to control parasites in their herd. Farmers gave reasons why medicinal plants are still in use, which included the efficacy of the plants, no side effects, and cheap and easy accessibility. Moreki, Dikeme and Poroga (2010) attributed the wide use of ethno-veterinary medicine in villages to lack of knowledge about the use of commercial drugs and their high price in most areas. However, Gueye (1999) argued that the use of ethno-veterinary medicine is the only option for most resource-limited farmers in Africa because of lack of veterinarians in the rural areas. Farmers reported that they were able to define diseases using clinical signs, but it should be kept in mind that some diseases exhibit similar signs (differential diagnosis) and that may affect the accuracy of the diagnosis. Wrong diagnosis of disease will result in incorrect dosage, and this can affect the efficacy of the herbal remedy.

The most frequently mentioned plant family was Asphodelaceae. Maphosa and Masika (2010) reported similar findings. This could be as a result of its vast natural distribution, with 13 genera (Treutlein *et al*. 2003). *Aloe ferox* and *Bulbine latifolia* were the plant species reported that belong to the family Asphodelaceae. In this study, leaves were the most used plant parts mentioned, which concurs with previous studies (Gebrezgabiher *et al*. 2013; Maphosa & Masika 2010; Masika & Afolayan 2003; Mohammed & Seyoum 2013; Setlalekgomo & Setlalekgomo 2013), who reported the highest percentage use of leaves for ethno-veterinary purposes. The use of leaves in preparation of herbal remedies reduces loss of plants from the natural habitats as it does not destroy the whole plant. On the other hand, this study contradicts findings by Cheikhyoussef *et al*. (2011), who reported that roots were the most commonly used plant part. The use of roots in preparation of herbal remedies is not advised, as this results in loss of medicinal plants from their habitats. Decoction was the most commonly used preparation method in this study. This is in agreement with Maphosa and Masika (2010) and Djoueche, Azebaze and Dongmo (2011), who reported decoction as the most used method for preparing medicine. Decoction involves boiling part of the plant in water for a few minutes. This process extracts water-soluble polar compounds and the high temperature is responsible for reducing the toxicity of thermolabile compounds that may be poisonous to the animals (Djoueche *et al*. 2011). However, some thermolabile compounds may also be lost in the process, which can affect the efficacy of the plant.

Some of the respondents combined more than one plant in preparation of medicines. The use of combined plant materials was also reported by Masika *et al*. (2000) and Maphosa and Masika (2010) but contradicts findings by Van der Merwe, Swan and Botha (2001), who reported use of a single plant. Other respondents mixed plant materials with nonplant substances such as Epsom salts, rock salt, butter, oil cake, flour and potassium permanganate. These findings were similar to previous reports of mixing with nonplant materials (Djoueche *et al*. 2011; Maphosa & Masika 2010; Mohammed & Berhanu 2011; Mohammed & Seyoum 2013). Mixing of plant materials with nonplant substances influences the absorption of compounds contained in the plants (Djoueche *et al*. 2011). Epsom salts are known to have a laxative effect. Oil cakes increase bile secretion, which promotes the solubilisation of non–water-soluble compounds (Djoueche *et al*. 2011). Rock salt has emulsifying properties assisting formation of stable emulsions in the gastrointestinal tract and therefore increasing the solubilisation of alkaline compounds in plant extracts, thus increasing their absorption (Djoueche *et al*. 2011). Addition of butter is known to improve the flavour and reduce the chances of animals vomiting (Mohammed & Seyoum 2013).

Farmers in the Eastern Cape Province are aware of the toxicity of some plants such as *A. oppositifolia* and therefore add more water to the herbal preparations and boil the plant material before administering it to the animals. They also mixed *A. oppositifolia* with other plant materials such as *A. ferox*. *Akocanthera oppositifolia* is toxic because of the cardiac glycosides it contains. This is in consonance with studies by Maphosa and Masika (2010), who reported the awareness of poisonous plants by farmers in the Eastern Cape Province. Adding large quantities of water before boiling results in the extract becoming more dilute and therefore the toxicity of the plant will be reduced. Boiling the plant extract also reduces toxicity by evaporating aromatic poisonous compounds.

Some of the plants that were being used by farmers to control parasites in this study area have been reported to possess pharmacologically active substances. Of the nine plants species used to control parasites in goats in Chris Hani District, *A. ferox* was the most frequently used plant as previously reported (Maphosa & Masika 2010; Moyo & Masika 2009; Setlalekgomo & Setlalekgomo 2013). The plant *A. ferox* has a laxative effect because of the presence of glycoside aloin (Steenkamp & Stewart 2007; Eloff & McGaw 2014). It is also known as an insect repellent; is used to treat heartwater and gallsickness (Van Wyk, Van Oudtshoorn & Gericke 2002), poultry diseases, sheep scab, and to control ticks in cattle (Moyo & Masika 2009). *Elephantorrhiza elephantina* is used to treat heartwater (Eloff & McGaw 2014; Luseba & Van der Merwe 2006; Van der Merwe *et al*. 2001) and used in goats to control helminths (Maphosa & Masika 2010; Okoli, Tamboura & Hounranghe-Adote 2010) and in humans for high blood pressure (Mathias-Mundy & McCorkle 1989). *Elephantorrhiza elephantina* possesses antibiotic properties (Van Wyk & Wink 2004; Van Wyk *et al*. 2002). It relieves inflammation in animals and is also used as a purgative (Cocks 2006). *Centella coriacea* contains triterpenoids that have antibiotic and purgative effects. *Agapanthus praecox* has been reported to contain saponins that have antibiotic, analgesic, laxative, anti-oedema, anti-inflammatory and immunoregulatory effects (Van Wyk *et al*. 2002). *Albuca setosa* is used in the management of diabetes mellitus (Oyedemi *et al*. 2011). *Acokanthera oppositifolia* has been reported to treat anthrax and tapeworms (Dold & Cocks 2001). FL values of *A. ferox, A. oppositifolia, A. setosa* and *E. elephantina* were high, showing that most people in the area prefer these plants and constantly use them in controlling parasites. In this study, the most cited plants had the highest FL, contrary to findings by Njoroge (2012). Trotter and Logan (1986) reported that plants that are constantly used by people in a certain area are more likely to contain bioactive substances. Validation of these plants is important so as to isolate the active compounds and produce drugs from these plants.

Some plant species had low FL values because some of the respondents did not know the preparation methods and dosages. Most of the informants acquired their knowledge from their elders, as reported by Mwale *et al*. (2005) and Mwale and Masika (2009). Ethno-veterinary knowledge is not written and is accepted orally from elders, analogous with earlier studies (Farooq *et al*. 2008; Giday, Asfaw & Woldu 2009). It is important to document ethno-veterinary knowledge so that it will not be lost and to increase the use of common plants. The bulk of plant matter is collected from the natural vegetation, which poses a major threat to its existence. Farmers should use plant parts such as leaves rather than the whole plant to prevent plants from becoming extinct (Maroyi 2012). Other respondents cultivate the plants in their gardens, and this is not recommended as it is believed that monoculture conditions do not trigger the production of secondary metabolites (Schippmann, Leaman & Cunningham 2003). Plants that are cultivated are believed not to possess power compared with wild plants. Therefore, it is advisable to use wild plants that grow under stress conditions and competition, as they possess secondary metabolites.

### Conclusion and recommendation

The study revealed nine plant species that are used to control parasites in goats in Chris Hani District Municipality. It also revealed that information about ethno-veterinary medicine in this area is mostly confined to older people and there is a danger that this knowledge can be lost before being passed on to other generations. Therefore, there is an urgent need to document these medicinal plants before the death of knowledgeable people in the study area. Further research should be carried out to assess the efficacy and safety of the plants mentioned, especially those with the highest FL.
